# Bus Riding as Amplification Mechanism for SARS-CoV-2 Transmission, Germany, 2021^1^

**DOI:** 10.3201/eid3004.231299

**Published:** 2024-04

**Authors:** Meike Schöll, Christoph Höhn, Johannes Boucsein, Felix Moek, Jasper Plath, Maria an der Heiden, Matthew Huska, Stefan Kröger, Sofia Paraskevopoulou, Claudia Siffczyk, Udo Buchholz, Raskit Lachmann

**Affiliations:** Robert Koch Institute, Berlin, Germany (M. Schöll, J. Boucsein, F. Moek, M. an der Heiden, M. Huska, S. Kröger, S. Paraskevopoulou, C. Siffczyk, U. Buchholz, R. Lachmann);; European Centre for Disease Prevention and Control, Stockholm, Sweden (M. Schöll, J. Boucsein, F. Moek);; Public Health Authority Main-Kinzig-Kreis, Hesse, Germany (C. Höhn, J. Plath)

**Keywords:** COVID-19, 2019 novel coronavirus disease, coronavirus disease, severe acute respiratory syndrome coronavirus 2, SARS-CoV-2, viruses, respiratory infections, zoonoses, outbreak, bus, schools, travel, whole-genome sequencing, Germany

## Abstract

To examine the risk associated with bus riding and identify transmission chains, we investigated a COVID-19 outbreak in Germany in 2021 that involved index case-patients among bus-riding students. We used routine surveillance data, performed laboratory analyses, interviewed case-patients, and conducted a cohort study. We identified 191 case-patients, 65 (34%) of whom were elementary schoolchildren. A phylogenetically unique strain and epidemiologic analyses provided a link between air travelers and cases among bus company staff, schoolchildren, other bus passengers, and their respective household members. The attack rate among bus-riding children at 1 school was ≈4 times higher than among children not taking a bus to that school. The outbreak exemplifies how an airborne agent may be transmitted effectively through (multiple) short (<20 minutes) public transport journeys and may rapidly affect many persons.

Several waves of COVID-19 have occurred in Germany (*1*). After low incidence rates in summer 2021, case numbers started to increase in late August because of the Delta variant (*2*). During the fourth COVID-19 wave in mid-September 2021 (*1*), a local public health authority (PHA) noticed an unusual increase in SARS-CoV-2 infections involving local schools in and around a city within the state of Hesse. The 7-day incidence of SARS-CoV-2 infection in this district reached 103 on September 15, compared with 99 in Hesse and 84 nationwide (Robert Koch Institute database, unpub. data). State legislation at the time prescribed mandatory use of medical masks in various settings, quarantine for persons with a positive test result (with exemptions for persons vaccinated or previously infected with SARS-CoV-2), and access bans to certain facilities for persons with COVID-19–compatible signs/symptoms (*3*). In the local elementary school, 1 case each was detected in calendar weeks (CW) 35 and 36, but in 2 subsequent days (September 14 and 15) in CW 37, test results for 18 students were positive. Because many of the students were riding a school bus, the hypothesis of the potential role of bus riding drew public attention.

On September 30, 2021, local and state PHAs invited the Robert Koch Institute (RKI), Germany’s national public health institute, to jointly investigate the outbreak with the following 3 objectives: to describe the outbreak, its emergence and control; to identify potential sources and chains of transmission through a combination of epidemiologic investigations and whole-genome sequencing (WGS); and to investigate the risk, preventive factors, and modes of transmission, in particular with regard to the role of bus riding. We subsequently explored the role of bus transportation for SARS-CoV-2 transmission and the value of genomic data as a tool for identifying transmission chains.

## Methods

### General Outbreak Description

We defined an outbreak case-patient as anyone with a SARS-CoV-2 infection confirmed by reverse-transcription PCR (RT-PCR) who resided in the affected district; whose symptom onset (or their test date) was from September 6 through October 15, 2021; and whose SARS-CoV-2 sample was assigned to the outbreak strain (sublineage AY.103) or who had an epidemiologic link to a case-patient with the outbreak strain. To describe the outbreak, we analyzed epidemiologic data from notified case-patients with SARS-CoV-2 infection and data from the respective laboratory samples. The principal of the most affected elementary school provided us with data on hygiene and testing routines, lists of class members and test results, and information on potential alternative exposures in the school setting.

### WGS Analyses

We contacted laboratories to secure remaining specimens for WGS analysis from case-patients with potential links to the outbreak. In addition, we performed WGS on randomly selected specimens from case-patients from the greater region (*4*,*5*).

We used the complete-genome information to detect additional sequences that could belong to this outbreak cluster. For that purpose, we used the tool breakfast (https://github.com/rki-mf1/breakfast), which computes the pairwise genetic distance between multiple sequences. We assigned genomes with a genomic distance of <3 single-nucleotide polymorphisms from an isolate from another case-patient within the outbreak to the outbreak strain. The genomes used for the analyses were not sequenced in house but were obtained from the Germany nationwide integrated genomic surveillance of SARS-CoV-2 (*6*). Thus, sequences were assembled by the individual laboratories according to their own protocols before being submitted to the RKI as consensus sequences. Genomic surveillance of SARS-CoV-2 is based on a legal framework, which was established in the beginning of 2021. Within the molecular surveillance of SARS-CoV-2, samples were either randomly chosen (random samples) or selected according to prior defined criteria (targeted samples). From January 2021 through December 2021, >475,000 SARS-CoV-2 sequences were submitted, ≈40% of which were randomly selected. To reduce bias in the estimation of circulating virus variants and sublineage proportions, we used only random samples. To detect additional sequences within the outbreak cluster, we used all submitted sequences for similarity analysis.

### Placing the Outbreak Genomes in a Phylogenetic Tree of Global SARS-CoV-2 Genomes

To identify whether the outbreak originated from a single source and whether it was effectively contained, we placed SARS-CoV-2 sequences from the outbreak case-patients in a global phylogenetic tree. The global tree contains all sequences from the GISAID (https://www.gisaid.org), GenBank, COVID-19 Genomics UK Consortium (https://www.cogconsortium.uk), and China National Center for Bioinformation (https://www.cncb.ac.cn) databases as of July 16, 2023 (>15 million sequences), and we conducted the placement by using a tool that is part of the National Center for Biotechnology Information Genome Browser (https://genome.ucsc.edu/cgi-bin/hgPhyloPlace), which relies on UShER (*7*) for placement of new sequences. Because RKI uploads all SARS-CoV-2 sequences to both GISAID and GenBank, the National Center for Biotechnology Information tree contains duplicates, which we removed manually. We then plotted the resulting deduplicated trees in R (The R Foundation for Statical Computing, https://www.r-project.org) by using the packages ggtree, treeio, ggplot2, ape, and dplyr (*8*–*12*) (Appendix Table).

### Bus-Riding Role and Associated Risk/Preventive Factors

We interviewed employees of the bus company and asked about bus schedules, hygiene, testing practices, and technical specifications of the bus. Using a computer-assisted standardized survey, we conducted phone interviews addressing households with outbreak case-patients as well as nonaffected households. The survey was designed to investigate several hypotheses regarding the COVID-19 outbreak at the elementary school, including exposure at school, during school bus rides, and during other social contacts.

To define the most likely infection setting of the case-patients, we combined information from the informal interviews, the survey, and the surveillance system. Secondary case-patients who lived in the same household as an outbreak case-patient were assigned to the infection setting “household.” Only case-patients who rode the bus on days when the bus driver was assumed to be infectious were assigned to the infection setting “bus.” Members of the bus company were assigned to the infection setting “workplace.” Case-patients who took part in social events that were attended by outbreak case-patients were assigned to the infection setting “other.” Whenever information regarding the use of the affected bus was insufficient, we conservatively assumed a different infection setting. We first conducted a cohort study of all students in grades 1–4 and defined case-patients as students having a SARS-CoV-2 infection confirmed by RT-PCR and symptom onset (alternatively test date) during September 6–October 15, 2021. We defined all other students at this school as non–case-patients. We calculated contingency tables, attributable fractions, and risk ratios.

To calculate risk and protective factors during school bus transportation, we conducted a second cohort study of elementary school students who used the school bus >1 time during September 9–17, 2021. Case-patients in this cohort were defined as students with a SARS-CoV-2 infection confirmed by RT-PCR whose symptom onset (alternatively test date) was during September 14–23, 2021. Information on seating position, mask use, vaccination status, and cumulative time at risk (i.e., time spent on the bus) were compared for case-patients versus non–case-patients by using univariable logistic regression.

The outbreak investigation was conducted under the official mandate of the local PHA resulting from paragraph 25 section 1 sentence 1 German Infection Protection Act (IfSG) and was thus exempt from review ethics committee review. We followed local and RKI ethics and data protection standards.

## Results

### Outbreak Description

We identified 191 case-patients (median age 17.5 years; 50% female and 50% male), of which 65 (34%) were elementary schoolchildren. According to the criteria of the German Standing Committee on Vaccination, 23% of all outbreak case-patients were fully vaccinated (Table 1) (*13*).

**Table 1:** Main characteristics of case-patients in COVID-19 outbreak, by age group, Hesse, Germany, 2021*

Characteristic	Age, y						Overall no. (%), n = 191	
	<6, no. (%), n = 11	6–10, no. (%), n = 73	11–17, no. (%), n = 28	18–40, no. (%), n = 20	41–60, no. (%), n = 30	>60, no. (%), n = 29		
Sex								
F	5 (45.5)	32 (43.8)	14 (50.0)	14 (70.0)	13 (43.3)	18 (62.1)	96 (50.3)	
M	6 (54.5)	41 (56.2)	14 (50.0)	6 (30.0)	17 (56.7)	11 (37.9)	95 (49.7)	
Setting								
Household	7 (63.6)	5 (6.8)	18 (64.3)	19 (95.0)	27 (90.0)	9 (31.0)	85 (44.5)	
Bus	3 (27.3)	46 (63.0)	3 (10.7)	0	1 (3.3)	6 (20.7)	59 (30.9)	
School	0	19 (26.0)	1 (3.6)	0	0	0	20 (10.5)	
Workplace	0	0	0	0	2 (6.7)	4 (13.8)	6 (3.1)	
Travel	0	0	0	0	0	5 (17.2)	5 (2.6)	
Other	1 (9.1)	0	2 (7.1)	1 (5.0)	0	4 (13.8)	8 (4.2)	
Unknown	0	3 (4.1)	4 (14.3)	0	0	1 (3.4)	8 (4.2)	
Prior SARS-CoV-2 infection								
Yes	0	1 (1.4)	0	0	0	0	1 (0.5)	
No	11 (100)	72 (98.6)	28 (100)	20 (100)	30 (100)	29 (100)	190 (99.5)	
Symptomatic infection								
Yes	7 (63.6)	62 (84.9)	23 (82.1)	20 (100)	26 (86.7)	22 (75.9)	160 (83.8)	
No	4 (36.4)	11 (15.1)	5 (17.9)	0	4 (13.3)	6 (20.7)	30 (15.7)	
Value missing	0	0	0	0	0	1 (3.4)	1 (0.5)	
Severity of disease, most severe status selected								
No treatment	9 (81.8)	58 (79.5)	14 (50.0)	12 (60.0)	17 (56.7)	15 (51.7)	125 (65.4)	
Outpatient treatment	0	6 (8.2)	0	3 (15.0)	5 (16.7)	0	14 (7.3)	
Inpatient treatment	0	0	1 (3.6)	1 (5.0)	0	3 (10.3)	5 (2.6)	
Treatment in ICU	0	0	0	0	2 (6.7)	0	2 (1.0)	
Deceased	0	0	0	0	0	4 (13.8)	4 (2.1)	
Value missing	2 (18.2)	9 (12.3)	13 (46.4)	4 (20.0)	6 (20.0)	7 (24.1)	41 (21.5)	
COVID-19 vaccination status								
Fully vaccinated	0	0	0	10 (50.0)	14 (46.7)	19 (65.5)	43 (22.5)	
Not or not fully vaccinated	11 (100)	73 (100)	28 (100)	10 (50.0)	16 (53.3)	10 (34.5)	148 (77.5)	

*The variable on severity of disease combines information from the routine surveillance system with survey results, resulting in a high number of missing values. ICU, intensive care unit.

**Figure 1:**
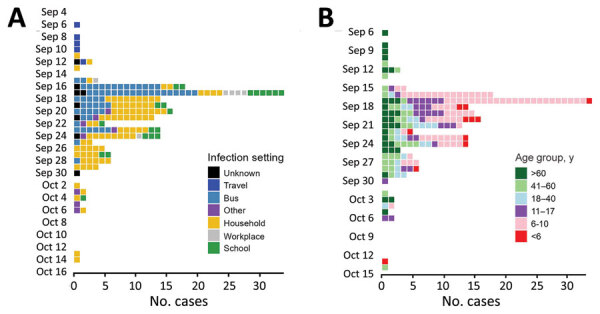
Epidemic curves for 191 case-patients in COVID-19 outbreak, Hesse, Germany, 2021. Dates are for symptom onset or first positive test result, whichever was earlier. A) By probable transmission setting; B) by age group.

Most (160 [84%]) case-patients were symptomatic, 5 (3%) were hospitalized, and 4 (2%) (>60 years of age) died. We identified only 1 SARS-CoV-2 reinfection. Case numbers associated with this outbreak peaked on September 17, 2021, and the outbreak was over by October 15, 2021 (Figure 1).

### Suboutbreak I among Elementary School Students

The most affected elementary school comprised 4 grade levels, each accommodating students of a similar age, with 3 to 4 classes (A, B, C, and D) per grade. Of 306 students, 70 were positive for SARS-CoV-2 during the investigation period, 65 of whom fulfilled the definition of outbreak case-patient (Table 2). Many students in classes A and B rode the school bus, and individual bus rides lasted 9–18 minutes. Students in classes C and D generally did not ride the school bus. In September 2021, students at the school were regularly tested by antigen test 3 times per week. In addition, all quarantined students (mostly from classes A and B) were tested by RT-PCR 1 time during CW 38.

**Table 2:** COVID-19 attack rates by class type and grade at an elementary school, Hesse, Germany, 2021

Class type or grade	No. students	No. COVID-19 case-patients	COVID-19 attack rate, %
Class			
A	88	43	49
B	91	25	28
C	84	2	2,4
D	43	0	0
Grade			
1	91	21	23
2	68	19	28
3	81	19	24
4	66	11	17

Results from the first cohort study show that 65 outbreak cases at the most affected school occurred in classes with bus-riding students, resulting in a relative risk of 24 (95% CI 6–95). Because testing practices differed between quarantined and nonquarantined students, cases in classes A and B were more likely to be detected, potentially biasing that relative risk. However, when we limited our analysis to students in classes A and B, the relative risk for bus-riding students was still 4 (95% CI 3–6) times greater than for those not riding the bus. That finding suggests that an attributable proportion of case-patients among students in classes A and B (74%) were exposed to SARS-CoV-2 by bus riding. Apart from bus riding, we did not identify any other common factor (e.g., social activities, common teacher, shared meals) among case-patients at that school.

In the second cohort study, among the 61 interviewed students who rode the school bus during September 9–17, a total of 45 students tested positive for SARS-CoV-2. Parents and care providers reported regular wearing of masks and variable seating arrangements for those children. Because the number of bus rides differed, cumulative time on the bus varied from 36 to 234 minutes. We found no statistically significant risk or protective factors among bus-riding students.

### Bus Driver and Bus Properties

The school bus driver tested positive for SARS-CoV-2 on September 15. As a close contact of a SARS-CoV-2–infected person and lacking vaccination, starting September 13, he was required to be quarantined. We were told that he instead drove the public bus, the school bus, and 1 charter tour bus (with female day-trippers) until September 15. In addition, several bus passengers (or their parents) reported that the bus driver was coughing and not wearing a mask for several days until he tested positive on September 15.

The obligation to wear masks during the ride was not generally enforced on the bus. The bus provided fresh air through air conditioning and regular air circulation; the 2 roof openings were not regularly opened in September.

### Tourist Group

The school bus driver also picked up 17 air travelers returning from a trip to Georgia on September 8, 2021. He transported the group for 1 hour. In early September, 6 of the travelers tested positive for SARS-CoV-2, 5 of whom fulfilled the definition of an outbreak case-patient (Figure 1). The first documented outbreak case-patient belonged to that tourist group, and symptoms had developed on September 6, 2021, while they were still traveling abroad.

### Public Passengers and Bus Company Staff

The bus driver also transported passengers on the public bus who were mostly adults and secondary school students, whereas passengers on the school bus were limited to students of 1 elementary school and 1 childcare center. We also identified subsequent case-patients among the driver’s close contacts at the bus company and in all of the described passenger groups. Of the 7 members of the bus company and their respective household members who had regular and close contact with each other, 6 tested positive over the course of the outbreak.

### Suboutbreak II among Day-Trippers

On September 15, 2021, the bus driver transported a group of 30 fully vaccinated female day-trippers, among which 6 became ill after the day trip. According to 1 woman’s recollection, none of the passengers wore a mask during the bus ride. The bus driver joined the group during lunch time. Two of the subsequent case-patients among the day-trippers sat directly behind the bus driver during the ride; a third case-patient shared a table with the bus driver during lunch. Symptoms developed in the case-patients 4–10 days after the day trip, and they tested positive 7–17 days after the trip. Alternative exposures, other than contact with the bus driver, were not disclosed for any of the 6 case-patients.

### WGS Data and Phylogenetic Sequences

**Figure 2:**
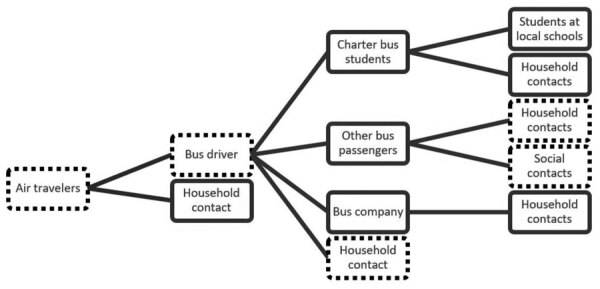
Plausible chains of transmission in COVID-19 outbreak in Hesse, Germany, 2021 (n = 191), Solid boxes indicate that whole-genome sequencing evidence of the outbreak strain was available for >1 case; dotted boxes indicate cases with no whole-genome sequencing evidence available.

**Figure 3:**
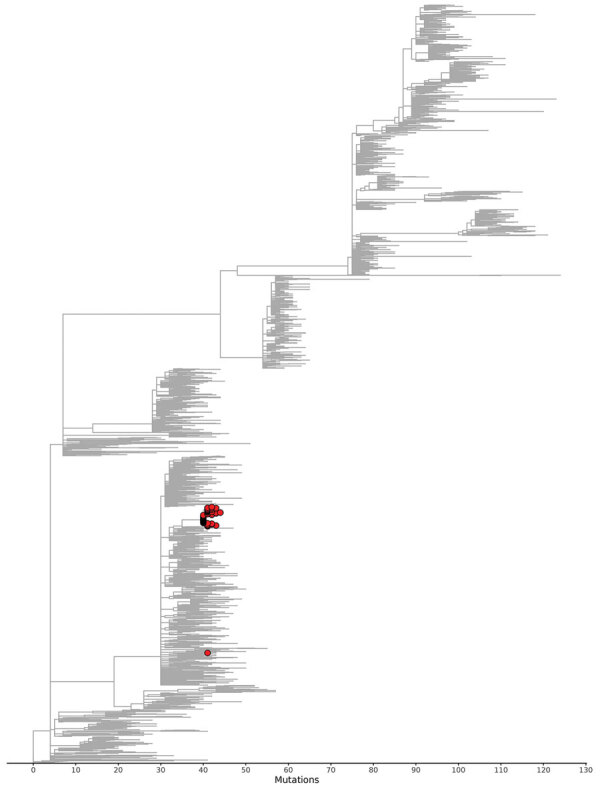
Sequences from samples from case-patients in COVID-19 outbreak, Hesse, Germany, 2021 (red circles). Sequences cluster tightly together when placed in a global phylogenetic tree, with the exception of 1 outlier. The outlier sequence from 1 air traveler was removed from the outbreak case-patients on the basis of lack of sequence similarity compared with other outbreak sequences. The global phylogenetic tree includes all SARS-CoV-2 sequences GISAID (https://gisaid.org), GenBank, COVID-19 Genomics UK Consortium (https://www.cogconsortium.uk), and the China National Center for Bioinformation (https://www.cncb.ac.cn) databases as of July 16, 2023 (total ≈15 million sequences) and was downsampled to ≈2,000 sequences for easier visualization.

**Figure 4:**
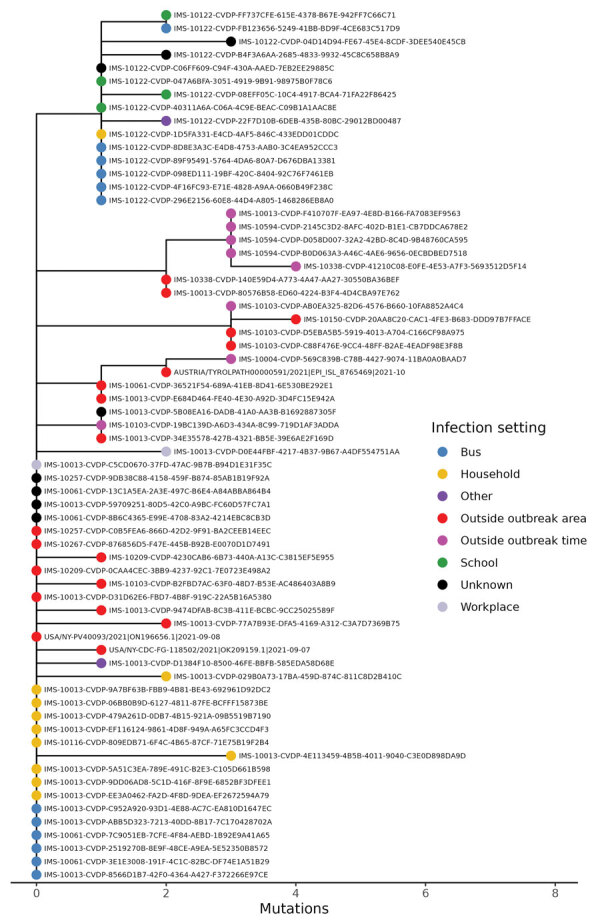
The subtree of the global SARS-CoV-2 phylogenetic tree in Figure 3 that contains all outbreak patient sequences contains few nonoutbreak sequences, showing that the outbreak in Hesse, Germany, 2021, was effectively contained. Three international sequences are included (2 from the United States, 1 from Austria), as well as 8 sequences that are from outside of the defined outbreak time period and 16 from outside the geographic region but still within Germany.

We had WGS data for 39 outbreak case-patients, including a secondary case-patient in the household of a returning traveler from Georgia. On the basis of the WGS data, we identified epidemiologic links for 31 case-patients with the outbreak strain; for the remaining 8, we did not have sufficient information. The phylogenetically unique strain (novel in Germany at this time) enabled us to link the following case-patients or case groups (1 case-patient from the tourist group with a previously established epidemiologic link was later excluded on the basis of sequencing findings): schoolchildren (all 15 available sequences belonged to the outbreak strain and 7 available sequences from household members of students from this school); the tourist group (1 sequence belonging to the outbreak strain from a household member of a traveler); the female day-trippers (1 available sequence belonged to the outbreak strain); subsequent case-patients among staff of the bus company (2 available sequences of the household members of the staff of the bus company belonged to the outbreak strain); and other bus passengers (transported by the same bus company), their respective household members, or both (Figure 1, panel B; Figures 2–4).

Using the national sequencing database at RKI, we compared the outbreak sequences not only to sequences obtained from the affected district but also to all sequences available for Germany. We identified 16 additional sequences in Germany and 3 sequences in Austria and the United States clustering with the outbreak sequence. The sequences from Germany belonged to case-patients outside of the district with symptom onset during September 21—October 12, 2021. Further epidemiologic information was available for 9 of those case-patients, all of whom could be epidemiologically linked to the outbreak; however, they were not included in the total case count because they resided outside the outbreak district.

## Discussion

Using integrated epidemiologic and genomic data, we documented the introduction of a novel SARS-CoV-2 strain (AY.103) to Germany by air travelers, leading to an outbreak. Our investigation also highlights effective transmission of the SARS-CoV-2 Delta variant on a bus. Most likely, at the time of arrival in Germany, the virus spread from the air travelers to the bus driver, who during the following days may have transmitted the virus to the bus-riding schoolchildren and other passengers. The outbreak spread further within the bus company, schools, and corresponding households. To our knowledge, further transmission was prevented because of measures such as testing and quarantine implemented by the PHA after outbreak detection.

Our investigation of suboutbreak I at the elementary school suggests that although the transit time among students was only 9–18 minutes twice a day, the attributable portion of early case-patients among students explained by bus riding amounts to 74%. The actual percentage might even be greater because we considered those case-patients to have been exposed only inside the charter bus, for which we were able to verify the information in interviews.

The suboutbreak II among day trippers who were exposed to the same bus driver as the schoolchildren provides further evidence regarding transmission chains. Nevertheless, we cannot determine whether transmissions took place only during the 1-hour bus ride or also while sharing a lunch table with the bus driver.

Given the delay between the timing of the outbreak and its investigation, most samples had already been disposed. However, data from the available samples agree with our epidemiologic findings and suggest transmission as described above. The sequence lineage had not been detected in Germany before the arrival of the tourist group, suggesting that the strain was introduced by the travelers. Although it seems unlikely that a person other than the bus driver might have served as the link between all the groups with the same outbreak strain (household person of an air traveler, bus company staff, bus riding children, day trippers), we cannot exclude the possibility of an unobserved additional transmission chain.

Regarding infection of the schoolchildren, transmission could have taken place somewhere other than inside the bus. Children in every school grade were affected, and the available sequences link the respective students or their household contacts or both to the outbreak cluster. In addition, analysis of the relative risks in the school classes showed a significantly elevated relative risk for infection among bus-riding students. Those observations strongly support the hypothesis that most infections among schoolchildren were associated with bus transportation. However, because most students rode the bus twice a day, it remains unclear if transmissions happened on a single bus journey or if the cumulative exposure over several days was key.

Epidemiologic and genomic data demonstrate that contact tracing, testing, isolation, and quarantine successfully contained the outbreak. Because the outbreak strain was unique within Germany, the phylogenetic analyses prove that the outbreak strain has not spread further. As such, the effectiveness of contact tracing, isolation, and quarantine in the middle of the wave of the extremely transmissible Delta variant is convincing.

WGS was key to validating the existing epidemiologic links and identifying additional case-patients outside the cluster. Furthermore, given the specific strain and broad sequencing data for strains from the area and globally, WGS helped confirm that we did not miss other suboutbreaks. The AY.103 strain was not detected in the area after the outbreak was over, which shows that the local transmission was contained.

Our findings are relevant given that evidence of SARS-CoV-2 transmission events associated with short bus rides or public bus transportation is limited (*14*). Rather, published transmission events or outbreaks associated with bus transportation stem from longer bus rides (*15*–*18*). A large cohort study in Norway early in the pandemic (when masks were not yet routinely worn) showed a dose–response relationship between using public transport and the risk of acquiring SARS-CoV-2 (*19*). A study of US students riding school buses with infected COVID-19 passengers yielded no secondary case-patients associated with bus transport (*20*). Lack of transmission may have resulted from thorough adherence to mask wearing, lower transmissibility, and susceptibility of children to the wild virus (compared with the Delta variant), or both (*21*; O.A. Uthman, unpub. data, https://www.medrxiv.org/content/10.1101/2022.08.26.22279248v1). Our article describes a high secondary attack rate on (multiple) short bus journeys of <20 minutes during circulation of the Delta variant.

Among the limitations of our findings, we note that because the outbreak investigation was retrospective, active case finding was limited and some cases might have gone unnoticed, particularly among vaccinated household contact persons who were asymptomatic and therefore not tested. Also, outbreak case-patients and their respective links to the outbreak may have been underascertained because of lack of epidemiologic or molecular evidence.

Recall bias may have affected our identification of epidemiologic links and the data quality. Given local media coverage, the outbreak and its potential links to bus transportation were stipulated before being properly investigated. To address that limitation, we applied a conservative approach toward epidemiologic links; instead of relying on school lists for use of bus transportation, we interviewed families about actual bus use and based confirmed bus transportation on those interviews only. However, some misattribution cannot be fully excluded, and the data quality regarding mask use and seat location within the bus remains limited, given the difficulty of recall and secondhand information from parents instead of the young children themselves.

Our report showcases how local bus transportation amplified a COVID-19 outbreak. In collaboration with the affected local institutions and bus company, the local PHA rapidly identified the ongoing COVID-19 outbreak and implemented successful measures to control it. Regular testing at school enhanced early detection of SARS-CoV-2 infections, and early quarantine helped minimize subsequent transmissions at school and shows the value of quarantine measures for nonvaccinated contact persons at that time. WGS was essential for proving transmission chains.

To identify and resolve respiratory virus superspreading events, use of public transport and multiple short (<20 minutes) bus rides should be considered as potential amplification mechanisms. PHA should also consider WGS as a useful early adjunct tool for outbreak investigations. Hygiene measures, such as regular testing and mask wearing, should be implemented indoors and on public transportation during similar epidemics or pandemics.

Appendix Additional information for study of bus riding as amplification mechanism for SARS-CoV-2 transmission by air travelers, Germany, 2021.
